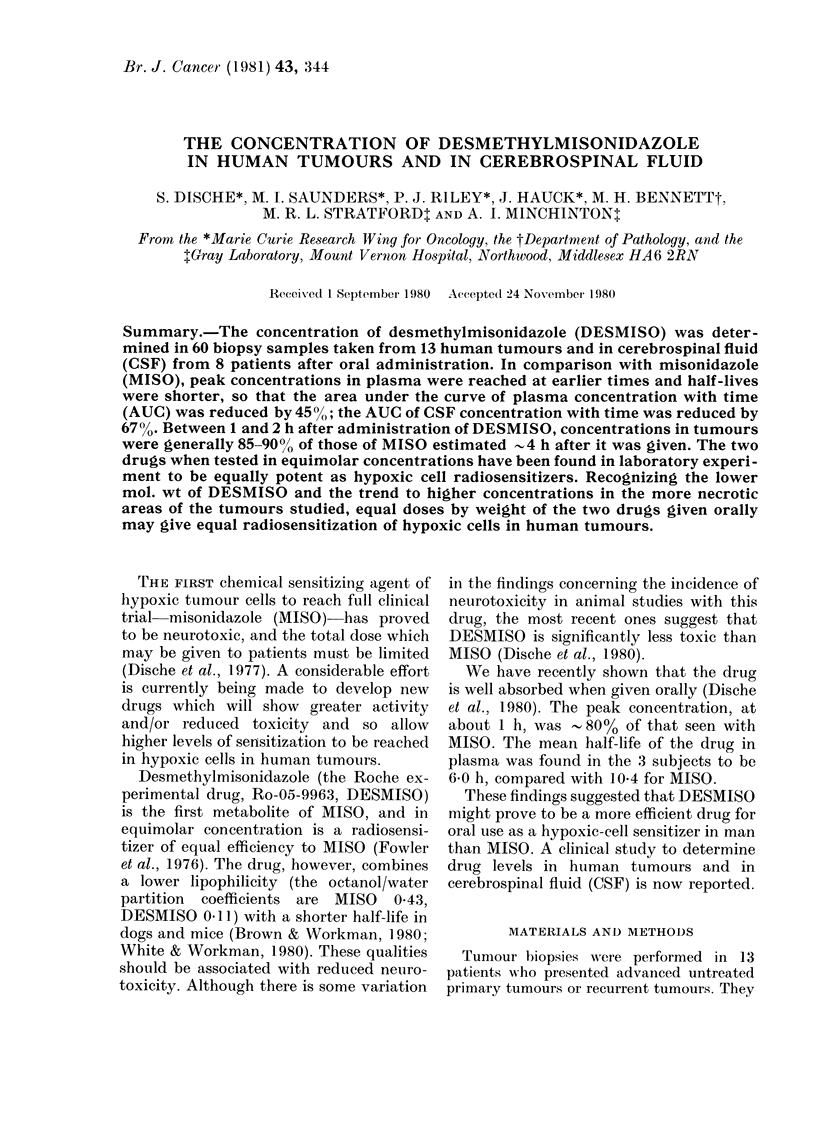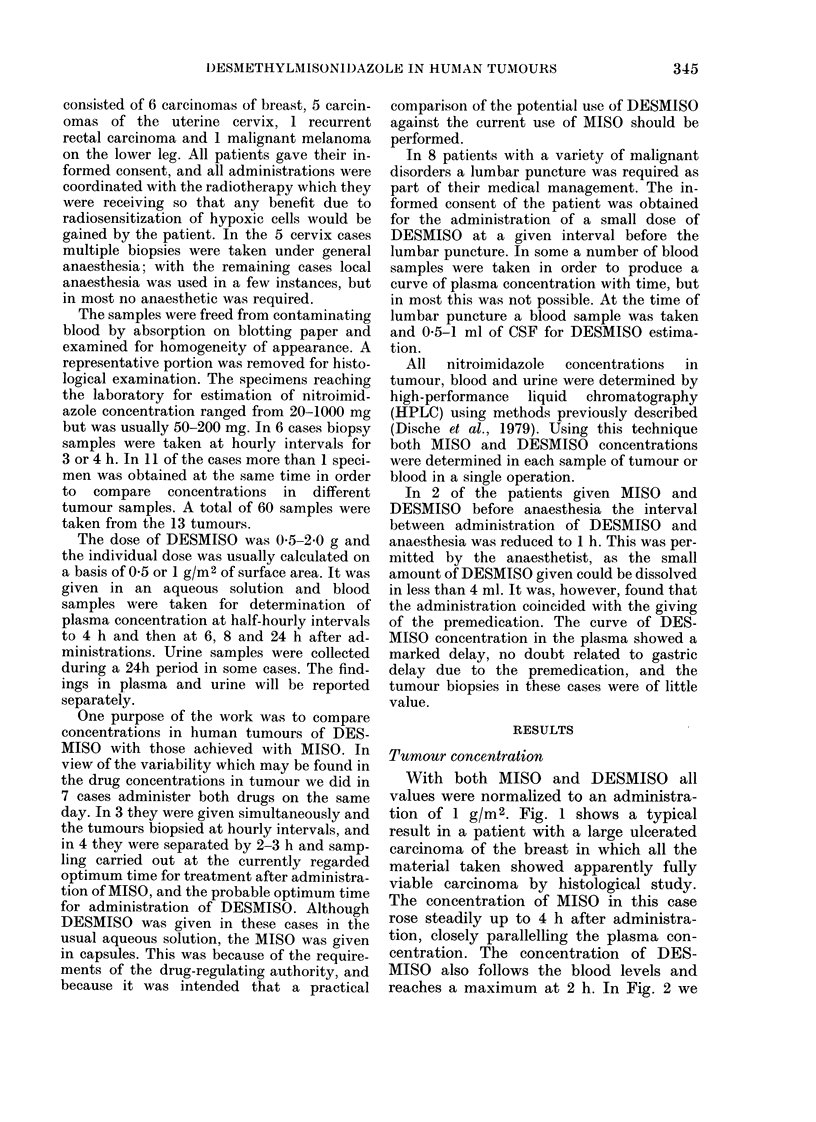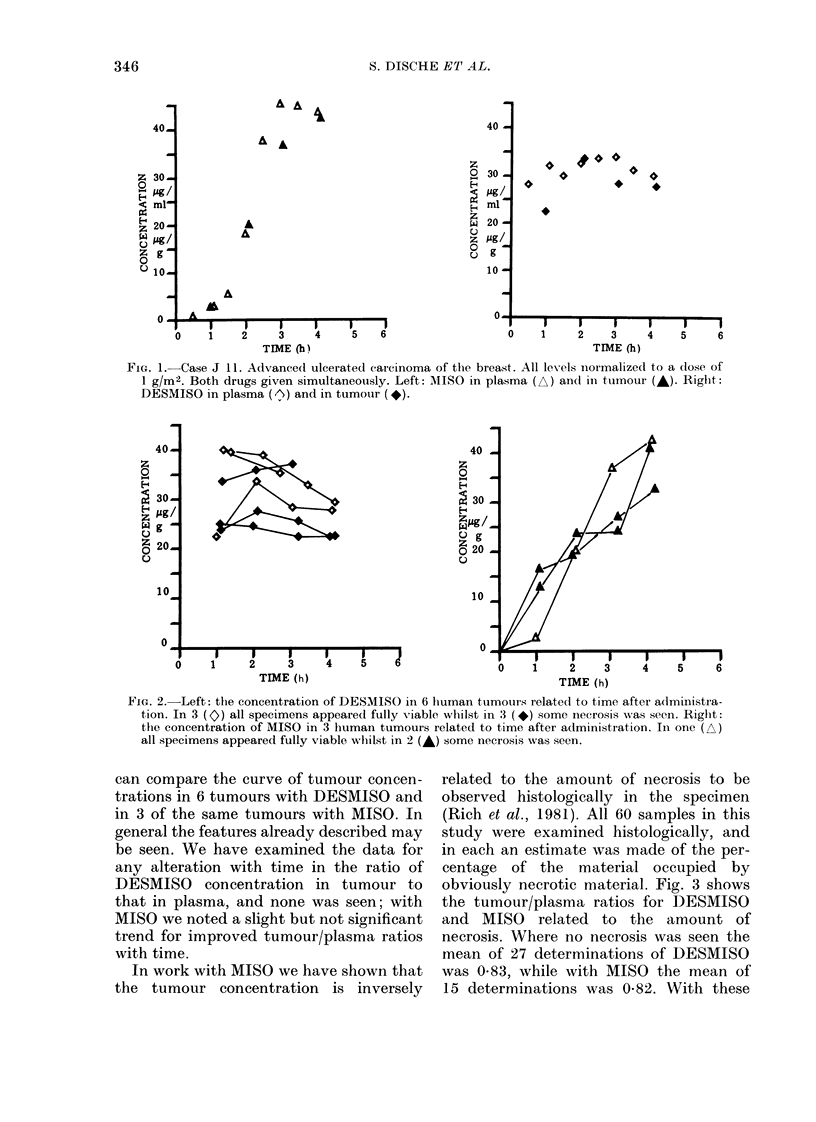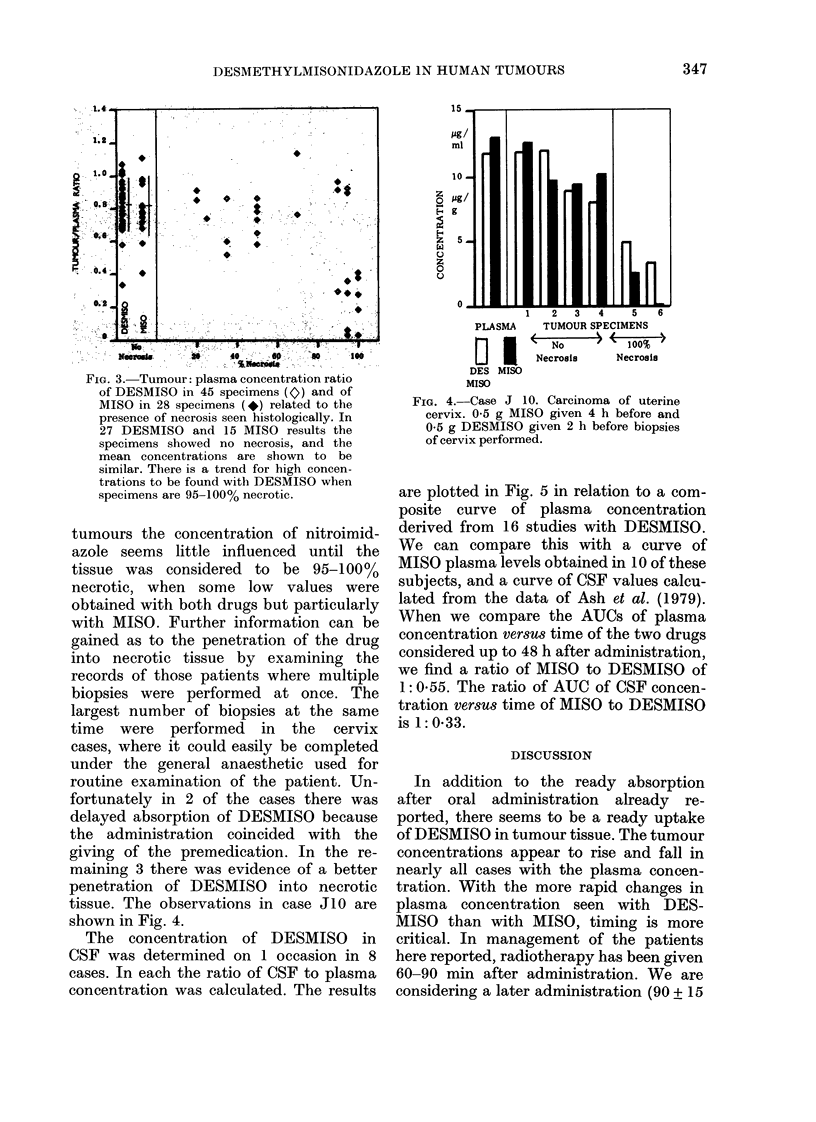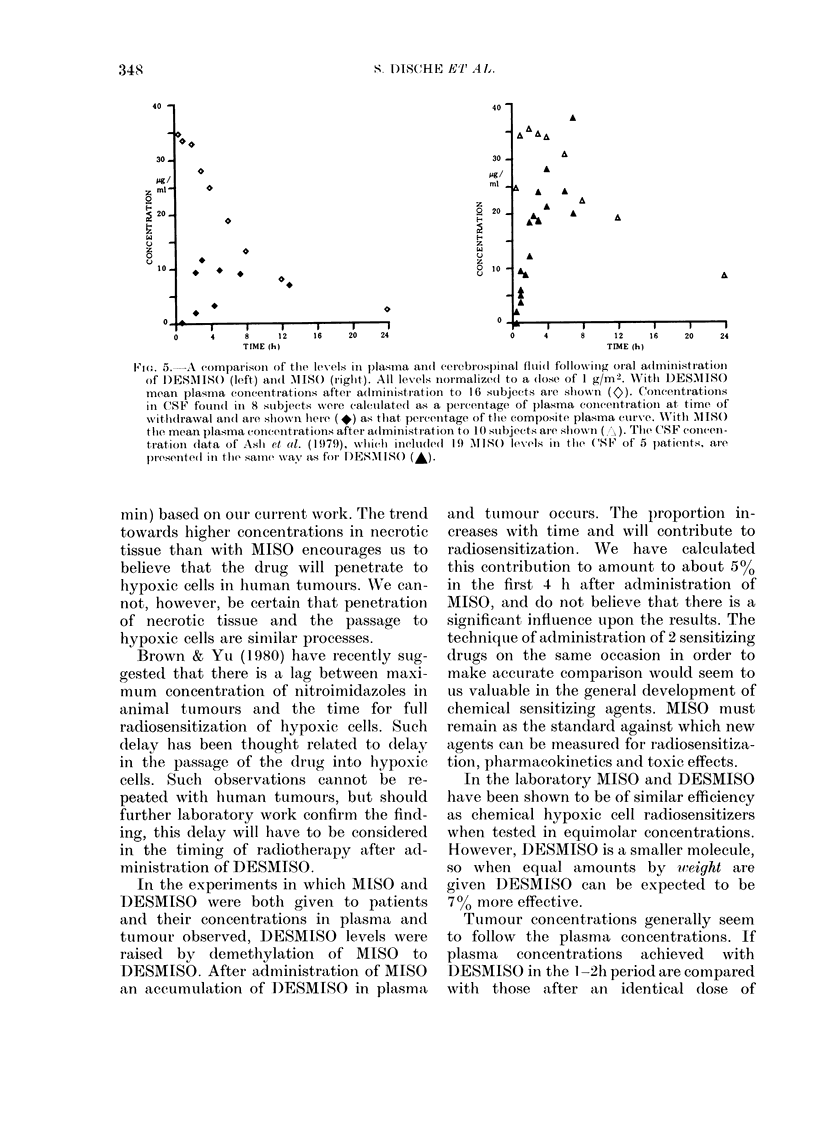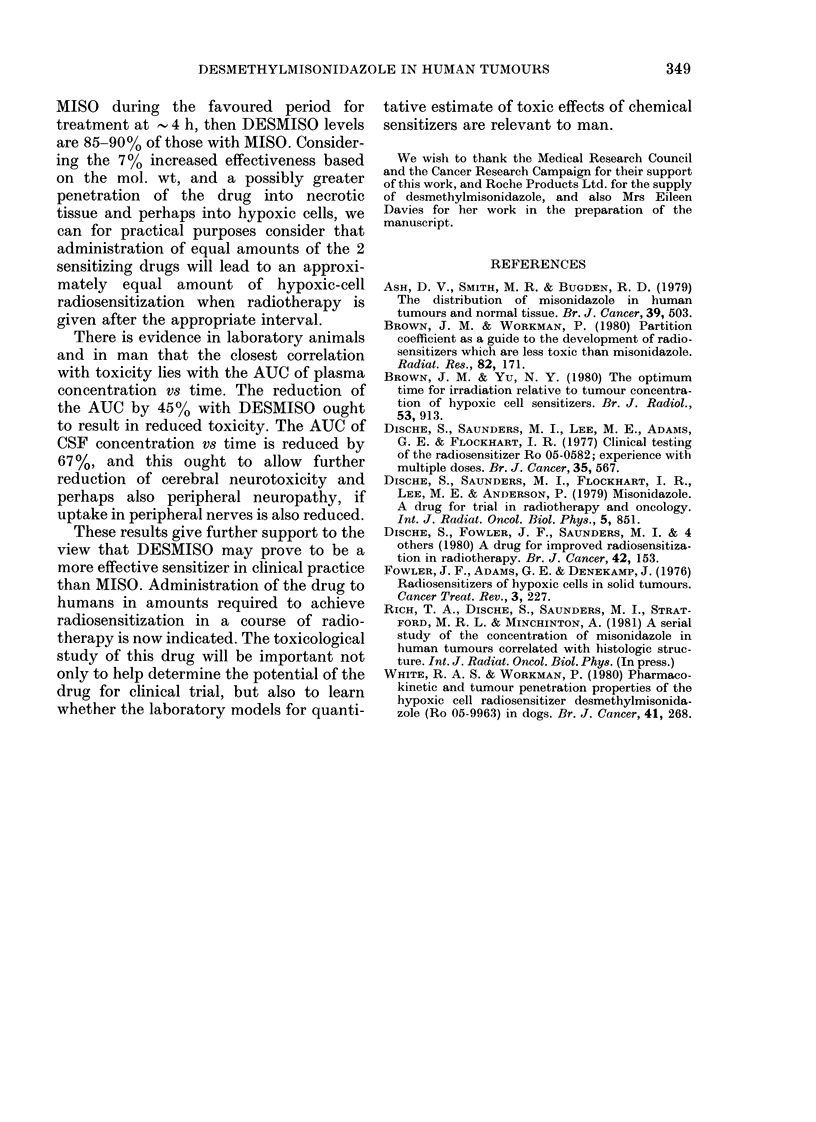# The concentration of desmethylmisonidazole in human tumours and in cerebrospinal fluid.

**DOI:** 10.1038/bjc.1981.54

**Published:** 1981-03

**Authors:** S. Dische, M. I. Saunders, P. J. Riley, J. Hauck, M. H. Bennett, M. R. Stratford, A. I. Minchinton

## Abstract

The concentration of desmethylmisonidazole (DESMISO) was determined in 60 biopsy samples taken from 13 human tumours and in cerebrospinal fluid (CSF) from 8 patients after oral administration. In comparison with misonidazole (MISO), peak concentrations in plasma were reached at earlier times and half-lives were shorter, so that the area under the curve of plasma concentration with time (AUC) was reduced by 45%; the AUC of CSF concentration with time was reduced by 67%. Between 1 and 2 h after administration of DESMISO, concentrations in tumours were generally 85-90% of those of MISO estimated approximately 4 h after it was given. The two drugs when tested in equimolar concentrations have been found in laboratory experiment to be equally potent as hypoxic cell radiosensitizers. Recognizing the lower mol. wt of DESMISO and the trend to higher concentrations in the more necrotic areas of the tumours studied equal doses by weight of the two drugs given orally may give equal radiosensitization of hypoxic cells in human tumours.


*Br. J. Cancer* (1981) **43**, 344

### THE CONCENTRATION OF DESMETHYLMISONIDAZOLE IN HUMAN TUMOURS AND IN CEREBROSPINAL FLUID

S. DISCHE*, M. I. SAUNDERS*, P. J. RILEY*, J. HAUCK*, M. H. BENNETT†, M. R. L. STRATFORD‡ AND A. I. MINCHINTON‡

*From the *Marie Curie Research Wing for Oncology, the †Department of Pathology, and the ‡Gray Laboratory, Mount Vernon Hospital, Northwood, Middlesex HA6 2RN*

Received 1 September 1980 Accepted 24 November 1980

**Summary.—The concentration of desmethylmisonidazole (DESMISO) was determined in 60 biopsy samples taken from 13 human tumours and in cerebrospinal fluid (CSF) from 8 patients after oral administration. In comparison with misonidazole (MISO), peak concentrations in plasma were reached at earlier times and half-lives were shorter, so that the area under the curve of plasma concentration with time (AUC) was reduced by 45%; the AUC of CSF concentration with time was reduced by 67%. Between 1 and 2 h after administration of DESMISO, concentrations in tumours were generally 85–90% of those of MISO estimated ~4 h after it was given. The two drugs when tested in equimolar concentrations have been found in laboratory experiment to be equally potent as hypoxic cell radiosensitizers. Recognizing the lower mol. wt of DESMISO and the trend to higher concentrations in the more necrotic areas of the tumours studied, equal doses by weight of the two drugs given orally may give equal radiosensitization of hypoxic cells in human tumours.**

THE FIRST chemical sensitizing agent of hypoxic tumour cells to reach full clinical trial—misonidazole (MISO)—has proved to be neurotoxic, and the total dose which may be given to patients must be limited (Dische *et al.*, 1977). A considerable effort is currently being made to develop new drugs which will show greater activity and/or reduced toxicity and so allow higher levels of sensitization to be reached in hypoxic cells in human tumours.

Desmethylmisonidazole (the Roche experimental drug, Ro-05-9963, DESMISO) is the first metabolite of MISO, and in equimolar concentration is a radiosensitizer of equal efficiency to MISO (Fowler *et al.*, 1976). The drug, however, combines a lower lipophilicity (the octanol/water partition coefficients are MISO 0·43, DESMISO 0·11) with a shorter half-life in dogs and mice (Brown & Workman, 1980; White & Workman, 1980). These qualities should be associated with reduced neurotoxicity. Although there is some variation in the findings concerning the incidence of neurotoxicity in animal studies with this drug, the most recent ones suggest that DESMISO is significantly less toxic than MISO (Dische *et al.*, 1980).

We have recently shown that the drug is well absorbed when given orally (Dische *et al.*, 1980). The peak concentration, at about 1 h, was ~80% of that seen with MISO. The mean half-life of the drug in plasma was found in the 3 subjects to be 6·0 h, compared with 10·4 for MISO.

These findings suggested that DESMISO might prove to be a more efficient drug for oral use as a hypoxic-cell sensitizer in man than MISO. A clinical study to determine drug levels in human tumours and in cerebrospinal fluid (CSF) is now reported.

### MATERIALS AND METHODS

Tumour biopsies were performed in 13 patients who presented advanced untreated primary tumours or recurrent tumours. They consisted of 6 carcinomas of breast, 5 carcinomas of the uterine cervix, 1 recurrent rectal carcinoma and 1 malignant melanoma on the lower leg. All patients gave their informed consent, and all administrations were coordinated with the radiotherapy which they were receiving so that any benefit due to radiosensitization of hypoxic cells would be gained by the patient. In the 5 cervix cases multiple biopsies were taken under general anaesthesia; with the remaining cases local anaesthesia was used in a few instances, but in most no anaesthetic was required.

The samples were freed from contaminating blood by absorption on blotting paper and examined for homogeneity of appearance. A representative portion was removed for histological examination. The specimens reaching the laboratory for estimation of nitroimidazole concentration ranged from 20–1000 mg but was usually 50–200 mg. In 6 cases biopsy samples were taken at hourly intervals for 3 or 4 h. In 11 of the cases more than 1 specimen was obtained at the same time in order to compare concentrations in different tumour samples. A total of 60 samples were taken from the 13 tumours.

The dose of DESMISO was 0·5–2·0 g and the individual dose was usually calculated on a basis of 0·5 or 1 g/m$^2$ of surface area. It was given in an aqueous solution and blood samples were taken for determination of plasma concentration at half-hourly intervals to 4 h and then at 6, 8 and 24 h after administrations. Urine samples were collected during a 24h period in some cases. The findings in plasma and urine will be reported separately.

One purpose of the work was to compare concentrations in human tumours of DESMISO with those achieved with MISO. In view of the variability which may be found in the drug concentrations in tumour we did in 7 cases administer both drugs on the same day. In 3 they were given simultaneously and the tumours biopsied at hourly intervals, and in 4 they were separated by 2–3 h and sampling carried out at the currently regarded optimum time for treatment after administration of MISO, and the probable optimum time for administration of DESMISO. Although DESMISO was given in these cases in the usual aqueous solution, the MISO was given in capsules. This was because of the requirements of the drug-regulating authority, and because it was intended that a practical comparison of the potential use of DESMISO against the current use of MISO should be performed.

In 8 patients with a variety of malignant disorders a lumbar puncture was required as part of their medical management. The informed consent of the patient was obtained for the administration of a small dose of DESMISO at a given interval before the lumbar puncture. In some a number of blood samples were taken in order to produce a curve of plasma concentration with time, but in most this was not possible. At the time of lumbar puncture a blood sample was taken and 0·5–1 ml of CSF for DESMISO estimation.

All nitroimidazole concentrations in tumour, blood and urine were determined by high-performance liquid chromatography (HPLC) using methods previously described (Dische *et al.*, 1979). Using this technique both MISO and DESMISO concentrations were determined in each sample of tumour or blood in a single operation.

In 2 of the patients given MISO and DESMISO before anaesthesia the interval between administration of DESMISO and anaesthesia was reduced to 1 h. This was permitted by the anaesthetist, as the small amount of DESMISO given could be dissolved in less than 4 ml. It was, however, found that the administration coincided with the giving of the premedication. The curve of DESMISO concentration in the plasma showed a marked delay, no doubt related to gastric delay due to the premedication, and the tumour biopsies in these cases were of little value.

### RESULTS

### *Tumour concentration*

With both MISO and DESMISO all values were normalized to an administration of 1 g/m$^2$. Fig. 1 shows a typical result in a patient with a large ulcerated carcinoma of the breast in which all the material taken showed apparently fully viable carcinoma by histological study. The concentration of MISO in this case rose steadily up to 4 h after administration, closely parallelling the plasma concentration. The concentration of DESMISO also follows the blood levels and reaches a maximum at 2 h. In Fig. 2 we

FIG. 1.—Case J 11. Advanced ulcerated carcinoma of the breast. All levels normalized to a dose of 1 g/m$^2$. Both drugs given simultaneously. Left: MISO in plasma (△) and in tumour (▲). Right: DESMISO in plasma (◇) and in tumour (◆).

FIG. 2.—Left: the concentration of DESMISO in 6 human tumours related to time after administration. In 3 (◇) all specimens appeared fully viable whilst in 3 (◆) some necrosis was seen. Right: the concentration of MISO in 3 human tumours related to time after administration. In one (△) all specimens appeared fully viable whilst in 2 (▲) some necrosis was seen.

can compare the curve of tumour concentrations in 6 tumours with DESMISO and in 3 of the same tumours with MISO. In general the features already described may be seen. We have examined the data for any alteration with time in the ratio of DESMISO concentration in tumour to that in plasma, and none was seen; with MISO we noted a slight but not significant trend for improved tumour/plasma ratios with time.

In work with MISO we have shown that the tumour concentration is inversely related to the amount of necrosis to be observed histologically in the specimen (Rich *et al.*, 1981). All 60 samples in this study were examined histologically, and in each an estimate was made of the percentage of the material occupied by obviously necrotic material. Fig. 3 shows the tumour/plasma ratios for DESMISO and MISO related to the amount of necrosis. Where no necrosis was seen the mean of 27 determinations of DESMISO was 0·83, while with MISO the mean of 15 determinations was 0·82. With these

FIG. 3.—Tumour: plasma concentration ratio of DESMISO in 45 specimens (◇) and of MISO in 28 specimens (◆) related to the presence of necrosis seen histologically. In 27 DESMISO and 15 MISO results the specimens showed no necrosis, and the mean concentrations are shown to be similar. There is a trend for high concentrations to be found with DESMISO when specimens are 95–100% necrotic.

FIG. 4.—Case J 10. Carcinoma of uterine cervix. 0·5 g MISO given 4 h before and 0·5 g DESMISO given 2 h before biopsies of cervix performed.

tumours the concentration of nitroimidazole seems little influenced until the tissue was considered to be 95–100% necrotic, when some low values were obtained with both drugs but particularly with MISO. Further information can be gained as to the penetration of the drug into necrotic tissue by examining the records of those patients where multiple biopsies were performed at once. The largest number of biopsies at the same time were performed in the cervix cases, where it could easily be completed under the general anaesthetic used for routine examination of the patient. Unfortunately in 2 of the cases there was delayed absorption of DESMISO because the administration coincided with the giving of the premedication. In the remaining 3 there was evidence of a better penetration of DESMISO into necrotic tissue. The observations in case J10 are shown in Fig. 4.

The concentration of DESMISO in CSF was determined on 1 occasion in 8 cases. In each the ratio of CSF to plasma concentration was calculated. The results are plotted in Fig. 5 in relation to a composite curve of plasma concentration derived from 16 studies with DESMISO. We can compare this with a curve of MISO plasma levels obtained in 10 of these subjects, and a curve of CSF values calculated from the data of Ash *et al.* (1979). When we compare the AUCs of plasma concentration *versus* time of the two drugs considered up to 48 h after administration, we find a ratio of MISO to DESMISO of 1: 0·55. The ratio of AUC of CSF concentration *versus* time of MISO to DESMISO is 1: 0·33.

### DISCUSSION

In addition to the ready absorption after oral administration already reported, there seems to be a ready uptake of DESMISO in tumour tissue. The tumour concentrations appear to rise and fall in nearly all cases with the plasma concentration. With the more rapid changes in plasma concentration seen with DESMISO than with MISO, timing is more critical. In management of the patients here reported, radiotherapy has been given 60–90 min after administration. We are considering a later administration (90 ± 15

FIG. 5.—A comparison of the levels in plasma and cerebrospinal fluid following oral administration of DESMISO (left) and MISO (right). All levels normalized to a dose of 1 g/m². With DESMISO mean plasma concentrations after administration to 16 subjects are shown (◇). Concentrations in CSF found in 8 subjects were calculated as a percentage of plasma concentration at time of withdrawal and are shown here (◆) as that percentage of the composite plasma curve. With MISO the mean plasma concentrations after administration to 10 subjects are shown (△). The CSF concentration data of Ash *et al.* (1979), which included 19 MISO levels in the CSF of 5 patients, are presented in the same way as for DESMISO (▲).

min) based on our current work. The trend towards higher concentrations in necrotic tissue than with MISO encourages us to believe that the drug will penetrate to hypoxic cells in human tumours. We cannot, however, be certain that penetration of necrotic tissue and the passage to hypoxic cells are similar processes.

Brown & Yu (1980) have recently suggested that there is a lag between maximum concentration of nitroimidazoles in animal tumours and the time for full radiosensitization of hypoxic cells. Such delay has been thought related to delay in the passage of the drug into hypoxic cells. Such observations cannot be repeated with human tumours, but should further laboratory work confirm the finding, this delay will have to be considered in the timing of radiotherapy after administration of DESMISO.

In the experiments in which MISO and DESMISO were both given to patients and their concentrations in plasma and tumour observed, DESMISO levels were raised by demethylation of MISO to DESMISO. After administration of MISO an accumulation of DESMISO in plasma and tumour occurs. The proportion increases with time and will contribute to radiosensitization. We have calculated this contribution to amount to about 5% in the first 4 h after administration of MISO, and do not believe that there is a significant influence upon the results. The technique of administration of 2 sensitizing drugs on the same occasion in order to make accurate comparison would seem to us valuable in the general development of chemical sensitizing agents. MISO must remain as the standard against which new agents can be measured for radiosensitization, pharmacokinetics and toxic effects.

In the laboratory MISO and DESMISO have been shown to be of similar efficiency as chemical hypoxic cell radiosensitizers when tested in equimolar concentrations. However, DESMISO is a smaller molecule, so when equal amounts by *weight* are given DESMISO can be expected to be 7% more effective.

Tumour concentrations generally seem to follow the plasma concentrations. If plasma concentrations achieved with DESMISO in the 1–2h period are compared with those after an identical dose of

MISO during the favoured period for treatment at $\sim 4$ h, then DESMISO levels are 85–90% of those with MISO. Considering the 7% increased effectiveness based on the mol. wt, and a possibly greater penetration of the drug into necrotic tissue and perhaps into hypoxic cells, we can for practical purposes consider that administration of equal amounts of the 2 sensitizing drugs will lead to an approximately equal amount of hypoxic-cell radiosensitization when radiotherapy is given after the appropriate interval.

There is evidence in laboratory animals and in man that the closest correlation with toxicity lies with the AUC of plasma concentration *vs* time. The reduction of the AUC by 45% with DESMISO ought to result in reduced toxicity. The AUC of CSF concentration *vs* time is reduced by 67%, and this ought to allow further reduction of cerebral neurotoxicity and perhaps also peripheral neuropathy, if uptake in peripheral nerves is also reduced.

These results give further support to the view that DESMISO may prove to be a more effective sensitizer in clinical practice than MISO. Administration of the drug to humans in amounts required to achieve radiosensitization in a course of radiotherapy is now indicated. The toxicological study of this drug will be important not only to help determine the potential of the drug for clinical trial, but also to learn whether the laboratory models for quantitative estimate of toxic effects of chemical sensitizers are relevant to man.

We wish to thank the Medical Research Council and the Cancer Research Campaign for their support of this work, and Roche Products Ltd. for the supply of desmethylmisonidazole, and also Mrs Eileen Davies for her work in the preparation of the manuscript.

### REFERENCES

ASH, D. V., SMITH, M. R. & BUGDEN, R. D. (1979) The distribution of misonidazole in human tumours and normal tissue. *Br. J. Cancer*, **39**, 503.

BROWN, J. M. & WORKMAN, P. (1980) Partition coefficient as a guide to the development of radiosensitizers which are less toxic than misonidazole. *Radiat. Res.*, **82**, 171.

BROWN, J. M. & YU, N. Y. (1980) The optimum time for irradiation relative to tumour concentration of hypoxic cell sensitizers. *Br. J. Radiol.*, **53**, 913.

DISCHE, S., SAUNDERS, M. I., LEE, M. E., ADAMS, G. E. & FLOCKHART, I. R. (1977) Clinical testing of the radiosensitizer Ro 05-0582; experience with multiple doses. *Br. J. Cancer*, **35**, 567.

DISCHE, S., SAUNDERS, M. I., FLOCKHART, I. R., LEE, M. E. & ANDERSON, P. (1979) Misonidazole. A drug for trial in radiotherapy and oncology. *Int. J. Radiat. Oncol. Biol. Phys.*, **5**, 851.

DISCHE, S., FOWLER, J. F., SAUNDERS, M. I. & 4 others (1980) A drug for improved radiosensitization in radiotherapy. *Br. J. Cancer*, **42**, 153.

FOWLER, J. F., ADAMS, G. E. & DENEKAMP, J. (1976) Radiosensitizers of hypoxic cells in solid tumours. *Cancer Treat. Rev.*, **3**, 227.

RICH, T. A., DISCHE, S., SAUNDERS, M. I., STRATFORD, M. R. L. & MINCHINTON, A. (1981) A serial study of the concentration of misonidazole in human tumours correlated with histologic structure. *Int. J. Radiat. Oncol. Biol. Phys.* (In press.)

WHITE, R. A. S. & WORKMAN, P. (1980) Pharmacokinetic and tumour penetration properties of the hypoxic cell radiosensitizer desmethylmisonidazole (Ro 05-9963) in dogs. *Br. J. Cancer*, **41**, 268.